# Screening for Early Signs of Paternal Perinatal Affective Disorder in Expectant Fathers: A Cluster Analysis Approach

**DOI:** 10.3390/jpm11010010

**Published:** 2020-12-23

**Authors:** Sonia Mangialavori, Michele Giannotti, Marco Cacioppo, Federico Spelzini, Franco Baldoni

**Affiliations:** 1 Department of Human Sciences, LUMSA University of Rome, 00193 Rome, Italy; m.cacioppo@lumsa.it; 2 Department of Psychology and Cognitive Sciences, University of Trento, 38068 Rovereto, Italy; michele.giannotti@unitn.it; 3 Department of Obstetrics & Gynecology, Hospital of Infermi of Rimini, 47923 Rimini, Italy; federico.spelzini@gmail.com; 4 Department of Psychology, University of Bologna, 40127 Bologna, Italy; franco.baldoni@unibo.it

**Keywords:** affective disorder, perinatal period, fatherhood, prevention, gender, screening

## Abstract

Previous studies documented gender-related differences in the expression of Perinatal Affective Disorders. However, little attention has been paid to screening the male population during the perinatal period. This study was based on three aims: (1) to investigate the mental health of expectant fathers based on their levels of depression, anxiety, addiction, anger attacks/hostility, and somatization, identifying psychological profiles; (2) to analyze the association between these profiles and the individual variable of perceived stress; (3) and to examine the association between these profiles and the couple’s variable of marital adjustment. A total of 350 Italian expectant fathers in the last trimester of pregnancy were asked to fill in questionnaires concerning perceived stress, dyadic adjustment, psychiatric symptomatology, and depression. Three different clusters were found: “psychologically healthy men” (68%) with low levels of symptoms on all the scales; “men at risk of externalized behavioral problems” (17.1%), characterized by one or more addictive or risky behaviors and moderate levels of scales scores; and “men experiencing psychological distress” (14.9%), with the highest scores on all the scales. A significant association emerged among the perceived stress, marital adjustment, and cluster membership. These results highlight the importance of screening fathers in perinatal health services, which are still predominantly mother-centered, and underscore the necessity to create tailored and personalized interventions.

## 1. Introduction

Although being a father for most men is a joyful and fulfilling journey [1], the transition to parenthood, or the arrival of an additional child, can also be perceived as overwhelming and demanding [2]. Indeed, it has been widely recognized that adjustment to fatherhood may negatively affect the men’s mental health, increasing psychological distress, depression, and anxiety from the prenatal period [3,4].

In the last decades, an ever-growing number of studies have addressed the impact of transition to parenthood on fathers’ mental health [5,6,7]; however, evidence to propose an appropriate gender-based screening for fathers is lacking [1,2,3,4,5,6,7,8]. In this regard, Walsh, Davis, and Garfield [9] highlighted the urgency of increased attention to screening for Paternal Perinatal Depression (PPND), stating that it is inappropriate to consider the identification, prevention, and treatment interventions of PPND as optional.

PPND is considered a specific disorder that many fathers may suffer from between pregnancy and the first year after childbirth. PPND is related to maternal perinatal depression [10,11,12] and poor outcomes in offspring, including externalizing and internalizing symptoms [13,14,15].

Several studies identified significant associations between PPND and some individual variables such as high levels of perceived stress [16,17], multiparity [2,18,19], having a previous history of psychiatric disorders [20], and experiencing stressful life events (e.g., job loss, divorce, mourning) [21,22]. Other studies have highlighted the positive correlation between PPND and risk of perinatal depression in their partners [23,24] and the negative association between PPND and marital adjustment [11,25,26].

Two recent meta-analyses showed a PPND prevalence in the world ranging from 8.4% [27] to 10.4% [23]. In addition, longitudinal studies have shown that pregnancy is a period of high risk for the onset of depressive symptoms in both expectant parents [19,28].

### 1.1. PPND Clinical Expression

According to the masked depression framework, PPND signs and clinical expression are different from those observed in Maternal Perinatal Depression (MPND), since men often exhibit externalizing symptoms defined as depressive equivalents to hide their depression condition [8,29]. In fact, depressive symptoms can be milder and less defined and are often comorbid with anxiety, somatic symptoms and complaints, hostility and/or anger attacks, substance use (alcohol and drugs), or other addictions or risky behaviors (e.g., gambling, compulsive use of computer/smartphone, or internet, driving very fast, extra marital affairs) [8,30,31]. For this reason, Baldoni [32] proposed to replace the term PPND with Paternal Perinatal Affective Disorder (PPAD) using a more inclusive definition to embrace the broad range of depressive symptoms related to male psychological perinatal distress. Clinicians treating men for depression have also confirmed, based on their clinical experience, that the men’s tendency to externalize their distress and provoke interpersonal conflict are “masculine-specific manifestations of depression” [33].

Since perinatal depression risks and psychological responses differ significantly based on gender [31,34,35], it would be helpful to consider the wide array of paternal affective symptoms. Thus, identifying fathers’ psychological distress profiles could help mental health professionals better recognize the condition of these men and to develop gender-sensitive screening tools and treatment options tailored to fathers.

### 1.2. Screening for Early Signs of PPND

Previous studies documented gender-related differences in the manifestation of perinatal depression, [31,36]; however, little attention has been paid to the screening practice in the male population, especially during the perinatal period [5,37]. However, during the occasional perinatal screening visits for expectant fathers, when participants are interviewed to assess if their symptomatology truly indicates depression, the researchers and clinicians use the Diagnostic and Statistical Manual of Mental Disorders(DSM) diagnostic criteria of five or more symptoms from the list of nine potential symptoms for depression [38]. These symptoms are identical for both men and women. Thus, to date, there is no acknowledgement in this diagnostic system that the two genders may experience and/or exhibit depression differently.

Although measures to assess male-type depressive symptomatology are available, such as the Gotland Male Depression Scale (GMDS) [39], they have not been specifically developed for the perinatal period. Indeed, research and screening of perinatal affective disorders are based almost exclusively on self-report scales that only consider symptoms associated with MPND. In this regard, recent findings highlighted several limitations of traditional scales in capturing paternal psychological distress.

For instance, even if the Edinburgh Postnatal Depression Scale (EPDS) [40] has been validated in fathers [41,42,43,44], there is not yet a shared consensus on the optimal cut-off scores for depression and anxiety, which change across studies. Moreover, Nishimura and Ohashi [45] revealed different rates of at-risk fathers using the CES-D (Center for Epidemiological Study Depression Scale) (7.5%; cut-off ≥ 16) and the EPDS (11.6%; cut-off ≥ 9). A Danish study [46] revealed that 20.6% of the at-risk fathers exceed the cut-off value on the GMDS but not on the EPDS. Similarly, Carlberg et al. [47] found that EPDS and GMDS were related to different risk factors and prevalence of PPND. Interestingly, a specific subgroup of fathers only showed externalizing symptomatology without conventional depressive symptoms, proving that a multidimensional and gender-based screening should be used to cover different clinical features of paternal perinatal distress. Considering these limitations, the number of at-risk fathers may be often underestimated, especially when the screening process does not include the assessment of male-type depressive symptoms. 

The analysis of different profiles of psychological distress during pregnancy has only been investigated in primiparous women [48]. In this study, three different profiles were found: (1) “psychologically healthy women” with low levels of symptoms of depression, anxiety and fear of childbirth; (2) “women experiencing pregnancy- and childbirth-related anxiety”, with an average state anxiety above the clinical value; and (3) “psychologically distressed women”, that included women who reported high levels of depressive and anxious symptoms, some above the clinical cut-offs. These findings underlined the importance of early psychological screening in order to understand the diverse experience of expectant parents and to develop person-centered interventions [48]. 

Hence, based on an integrative and gender-based perspective, the present study was based on three aims: (1) to investigate the mental health of expectant fathers based on their levels of depression, anxiety, addiction, anger attacks/hostility, and somatization by identifying psychological profiles; (2) to analyze the association between the emergent psychological profiles and the individual variable of perceived stress; and (3) to examine the association between these profiles and the couple’s dimension of marital adjustment.

## 2. Materials and Methods

### 2.1. Procedure and Participants

We initially recruited 423 expectant fathers. After this preliminary recruitment, 21 were excluded for not giving informed consent, 38 were excluded because they did not complete the questionnaire entirely, 9 were excluded because the participants had poor knowledge of Italian and, after a screening by the gynecologist, 14 were excluded because the partner had a pregnancy at risk. We decided to exclude those with a partner with a high-risk pregnancy because the literature highlights that these fathers may have greater psychological distress due to this partner’s condition [49,50].

In total, this cross-sectional study involved 350 Italian expectant fathers (Mean age = 35.63, Standard Deviation = 6.32, range = 20–58) in the last trimester of pregnancy. Participants were recruited at the OB/GYN Department of the “Infermi” hospital of Rimini, and of the “Santo Spirito” and San “Filippo Neri” hospitals of Rome where they attended antenatal classes or routine visits between 2016 and 2019. Expectant fathers were informed about the aims and methodology of the study before signing the written consent form. Informed consent was obtained from all subjects involved in the study. 

The study was conducted in accordance with the Declaration of Helsinki, and the protocol was approved by the Ethics Committee of Infermi Hospital (Nº 3691/2016).

Study inclusion criteria were being 18 years or older, in a de facto or marital relationship, and in the third trimester of pregnancy. Exclusion criteria were having a partner with a high-risk pregnancy defined as the presence of one or more maternal and/or fetal health problems including pregnancy-induced hypertension, multiple gestations, medical disorder complicating pregnancy (such as diabetes), previous miscarriages, chromosomal abnormalities in the fetus, pregnancy complications (such as abnormal placenta position, fetal growth restriction) and threatened premature labor; refusal to provide informed consent; presence of cognitive disability and/or current psychiatric diagnosis; poor knowledge of Italian, or other verbal communication limitations that compromised the participant’s ability to follow the research protocol.

### 2.2. Measures

The Center for Epidemiologic Studies Depression Scale (CES-D) [51] is a 20-item self-report measure used to assess depressive symptomatology in the last week measured on a 4-point Likert scale, ranging from 0 to 3. Summing responses to all items formed the depression score, with higher scores indicating more depressive symptoms. The CES-D has been used extensively in community settings and among expectant parents [52]. The Italian version of CES-D [53] was used in this study, showing a satisfactory level of internal consistency (α = 0.71).

The Symptom Checklist-90-Revised (SCL-90-R) [54] is a well-known 90-item questionnaire, scored on a Likert scale from 0 to 4, that is used to assess psychiatric symptomatology. In this study, Anxiety (ANX); Somatization (SOM); and Hostility (HOS) subscales were used, with higher scores indicating higher symptoms frequency. The Italian version of SCL-90-R [55] was used, showing a fair level of internal consistency for all the subscales respectively α = 0.72 for ANX, α = 0.78 for SOM, and α = 0.75 for HOS.

The Perceived Stress Scale (PSS) [56] was used to measure the perception of stress in the last six months. It is a measure of the degree to which situations in one’s life are appraised as stressful. It contains 10 items that are rated on a 5-point scale that ranges from never to very often. High total scores indicate greater perceived stress. The PSS was widely used during the perinatal period both for mothers and fathers [57]. In this study, the Italian validation [58] was used, showing a good level of internal consistency (α = 0.76).

The Dyadic Adjustment Scale (DAS) [59] was used to assess a couple’s functioning. It is composed of 32 items, 31 of which are related to the specific dimension of marital adjustment while one item refers to the overall perceived happiness with the relationship. In this study, the Italian validated version [60] showed a very good internal consistency (α = 0.89).

Addictions and other risky behaviors were assessed with ad hoc categorical (yes or no) item “In the previous two weeks, I smoked, drank alcohol, used drugs, gambled or used the internet more than usual; or I have taken risks more than usual (e.g., driving very fast, doing dangerous sports, unnecessary risks at work, etc.) (one or more of these)”.

Finally, Sociodemographic information (age, education, occupation, number of children) and individual information about the previous history of psychiatric disorders and the presence of stressful life events (e.g., job loss, divorce, mourning) in the previous six months were investigated.

### 2.3. Data Analysis

Data were analyzed using the Statistical Package for the Social Sciences, version 23 (SPSS Inc., Chicago, IL, USA) and are presented as means, standard deviations (SD), ranges and percentages (%). The correlation index between study variables (CES-D, ANX, SOM, HOS, PSS, and DAS) was calculated.

As suggested by Kent, Jensen and Kongsted [61], in order to identify different sub-groups of psychological distressed men characterized by high within-cluster homogeneity and high between-cluster heterogeneity, a Two-Step cluster analysis was performed on the continuous variables of CES-D, ANX, SOM, and HOS together with the categorical addiction/risky behaviors variable. 

The Two-Step cluster analysis is a statistical approach that first uses a distance measure to separate groups and then a probabilistic approach to select the optimal sub-group model [61]. Two-Step cluster analysis is also considered more reliable and accurate when compared to traditional clustering methods such as the k-means clustering algorithm [62,63]. This technique presents several advantages compared to more traditional techniques, such as determining automatically the number of clusters based on a statistical measure of fit (AIC or BIC) rather than on an arbitrary choice, using categorical and continuous variables simultaneously, analyzing atypical values (i.e., outliers), and being able to handle large datasets [61,64]. Comparative studies regarded Two-Step cluster analysis as one of the most reliable in terms of the number of subgroups detected, the classification probability of individuals to subgroups, and the reproducibility of findings on clinical data [61,65]. In the first step (pre-clustering), a sequential approach is used to pre-cluster the cases with the aim to reduce the size of the matrix that contains distances between all possible pairs of cases. In the second step (clustering), the pre-clusters are clustered using the hierarchical clustering algorithm. No prescribed number of clusters was suggested, and the log-likelihood criterion was used for distance measure. Schwarz’s Bayesian criterion (BIC) and the silhouette coefficient were used to compare cluster solutions. Silhouette measures of less than 0.2 were classified as poor; between 0.2 and 0.5 were classified as fair; and greater than 0.5 were classified as good solution quality, with fair or higher considered acceptable clustering [64]. 

Regarding the second and third aims of the study, the association among psychological profiles, perceived stress (PSS), and dyadic adjustment (DAS) was tested through two univariate ANOVAs with the Bonferroni correction in the post hoc tests. 

The level of statistical significance was set at *p* < 0.05.

Moreover, to provide a more comprehensive descriptive analysis, the association between psychological profiles and some individual variables (being or not a primiparous parent, previous psychiatric conditions, and the presence of stressful life events) was investigated through chi-square statistics with the standard residual method, as post hoc, to identify those specific cells making the greatest contribution to the chi-square test result [66]. In line with Field [67], since, in our case, the inspection of residuals was used as a guide to what cells might be of interest, we preferred to choose a more conservative alpha value than 0.05 such 0.01 (z value +/− 2.58).

## 3. Results

Descriptive variables of the study sample (sociodemographic characteristics, being or not a primiparous parent, previous psychiatric diagnosis, presence of stressful life events) are presented in Table 1. Descriptive statistics of the psychological dimensions (CES-D, ANX, SOM, HOS, PSS, DAS, addiction/risky behavior item) are presented in Table 2. All the variables were normally distributed. Correlation coefficients among the variables of interests are reported in Table 3. All the variables were significant for each cluster (Table 4). The composition of the clusters and the importance of variables within a cluster have been examined.

When we only consider the CES-D cut-off [51], the rate of men at risk of depression was 8.2% (*n* = 29; cut-off ≥ 16).

Regarding the SCL-90 mean scores, when we compared the mean scores of the subscales anxiety (ANX), somatization (SOM), and anger/hostility (HOS) to the Italian norms, only the anxiety mean score was higher than the general male population mean score, but it did not reach clinical significance (T < 45) [55].

With respect to the first aim of the study, the Two-Step cluster analysis yielded three clusters (BIC = 817.04; ratio of distance measure = 2.28), with no exclusion of cases. The Schwarz BIC was selected as the final clustering criterion because it provides a more precise cluster estimate [63] and the three-cluster solution provided a silhouette coefficient S(i) of 0.6, which indicates a good amount of separation and cohesion between data points within the clusters and overall goodness of fit cluster solution [64,68,69]. 

In term of predictive variables, depressive, anxious, and somatic symptomatology together with anger/hostility and addictive/risky behaviors were the five input variables for the generation of the clusters.

The first cluster included 68% of the total sample (*n* = 238), and it was characterized by low levels of anxiety, depression, hostility, somatization, and the absence of any reported addictive or risky behaviors. We defined it as a “psychologically healthy men” cluster. In the second cluster (14.9% of the study sample; *n* = 52), expectant fathers reported the higher scores for anxious and depressive symptoms, hostility as well as somatization, whereas the majority of them (*n* = 43, 82.7%) did not fit in the addictive and risky behaviors category. Thus, this cluster was named “men experiencing psychological distress”. The third cluster included 60 expectant fathers (17.1% of the total sample), and it comprised primarily the presence of one or more addictive or risky behaviors in the last two weeks with perceived anxiety, depression, hostility, and somatization represented to a moderate degree. We named this cluster as “men at-risk of externalized behavioral problems”. The ratio of sizes, largest cluster to smallest cluster, was 4.68. 

For the first cluster, anxious symptoms emerged as main predictor for the group membership with a predictor importance (PI) of 0.93, followed by hostility (PI = 0.50), somatization (PI = 0.49), addictive/risky behaviors (PI = 1.00), and depressive symptoms (PI = 0.38). For the second cluster, anxious symptoms emerged as the main predictor (PI = 0.93), followed by depressive symptoms (PI = 0.38), somatization (PI= 0.49), hostility (PI = 0.50), and addictive/risky behaviors (PI = 1.00). Considering the third cluster, the main predictor was addictive/risky behaviors dimension (PI = 1.00), followed by hostility (PI = 0.50), depressive symptoms (PI = 0.38), anxious symptoms (PI = 0.93), and somatization (PI = 0.49). 

According to our second aim, the findings revealed a significant association between cluster membership and perceived stress (F (2, 347) = 56.53, *p* < 0.001). In particular, perceived stress was significantly different between psychologically healthy men and psychologically distressed men, with men in the first cluster reporting an average score on PSS that was significantly lower than psychologically distressed men (mean difference = −7.52; standard error = 0.75; *p* < 0.001) and men at-risk of externalized behavioral problems (m.d. = −3.94; s.e. = 0.71; *p* < 0.001). Moreover, men in the second cluster obtained a higher average score on the PSS than men at-risk of externalized behavioral problems (m.d. = 3.58; s.e. = 0.93; *p* < 0.001).

Finally, as regards the third research aim, findings revealed a significant association between marital adjustment and cluster membership (F (2, 347) = 16.88, *p* < 0.001). Specifically, psychologically healthy men reported an average DAS score that is significantly higher than men at-risk of externalized behavioral problems (m.d. = 10.30; s.e. = 2.22; *p* < 0.001) and psychologically distressed men (m.d. = 9.05; s.e. = 2.09; *p* < 0.001); whereas no differences emerged between men at-risk of externalized behavioral problems and psychologically distressed men.

Regarding the descriptive analysis between the three emergent psychological profiles and individual variable of being or not a primiparous parent, the chi square test was not significant (χ^2^(2) = 1.44, *p* = 0.48). The association between the three clusters and the presence of previous psychiatric disorders was statistically significant (χ^2^(2) = 19.22, *p* < 0.01), while most of the individuals in the cluster of “psychologically healthy men” did not have previous psychiatric disorders (*n* = 220, 92.43%). The highest percentage of those who had previous psychiatric history was from individuals in the cluster of “psychologically distressed men” (*n* = 15, 28.84%), while the percentage of individuals who had previous psychiatric history of cluster of “men at-risk of externalized behavioral problems” was 16.66% (*n* = 10). A chi-square post-hoc test via the standard residual method confirmed that the standard residuals in the “psychologically healthy men” group category with previous psychiatric disorders significantly contributed to a significant omnibus chi-square statistic (χ^2^ = 15.37; *p* < 0.001). In addition, the inspection of standard residuals in the “psychologically distressed men” group category with the presence of previous psychiatric disorders significantly contributed to a significant omnibus chi-square statistic (χ^2^ = 15.52; *p* < 0.001), while it was observed that the standard residuals of “men at-risk of externalized behavioral problems” group with the variable of previous psychiatric disorders did not contribute to significant omnibus chi-square statistic (χ^2^ = 1.28; *p* = 0.77). 

Furthermore, the association between the three clusters and the presence of stressful life events was statistically significant (χ^2^(2) = 18.27, *p* < 0.01) with individuals of cluster “psychologically distressed men” had a higher percentage of negative past events than the other two groups (*n* = 31, 59.61%), whereas the men in the third cluster had a percentage of 31.66% (*n* = 19). Most of the men in the first cluster (66.80%; *n* = 159) had reported no presence of stressful life events in the previous six months. A chi-square post-hoc test via the standard residual method showed that only the standard residuals in the “psychologically distressed men” category with the stressful life events variable significantly contributed to significant omnibus chi-square statistic (χ^2^ = 13.59; *p* < 0.001). 

## 4. Discussion

The expression of father psychological distress during the perinatal period tends to be multifaceted compared to maternal depressive symptomatology, including a wide range of symptoms as depressive equivalents. Thus, the conventional self-report questionnaires used for the screening of perinatal depression in mothers may be not sufficient to capture paternal psychological distress during transition to parenthood. In particular, the manifestation of male-type symptoms may be overlooked, leading to an underestimation of at-risk fathers. Therefore, it becomes essential to consider depressive equivalents, especially externalizing behaviors, for the screening of early signs of PPND. To this purpose, the current study examined psychological distress profiles in expectant fathers, using a cluster-analysis approach and testing their associations with individual and couple dimensions. 

Firstly, the percentage of at-risk fathers in our sample is relatively in line with the rates of PPND emerged in previous studies [27,70]. Notably, we found that a greater number of fathers (32%) might be at-risk of developing a paternal affective disorder when other types of symptoms related to the expression of paternal perinatal distress were considered. Therefore, in these cases, a prevalence of depression in mothers and fathers can be similar, consistently with a previous study showing no differences between gender in rates of depression [31]. 

It has been argued that the underestimation of perinatal depression in men compared to women could be related to the type of measurements, which have been developed to address maternal mental health issues. This discrepancy highlighted the need to cover a wide range of clinical manifestations in fathers to address the impact of transition to fatherhood on paternal mental health [8,37]. 

Specifically, we found three profiles of paternal psychological distress during the prenatal period. The larger group included expectant fathers who reported lower levels of symptoms across the different investigated domains (anxiety, depression, hostility, and somatization). None of the expectant fathers of the “psychologically healthy men” reported addictive or risky behaviors during the last two weeks before the assessment. This finding confirms that most men perceived the transition to fatherhood as an adaptive process, without reporting specific symptoms of clinical significance during the screening process. 

Focusing on the at-risk groups, the third cluster of expectant fathers defined as “men at risk of externalizing behaviors’’ is characterized primarily for the manifestation of one of more addictive or risky behaviors during the third trimester of pregnancy. Thus, expectant fathers may feel the need to express their psychological distress reacting with externalizing symptoms such as substance use, gambling, internet addiction, self-disruptive, and other risky behaviors as highlighted by previous research [1,31,37]. A possible explanation is that the adherence to traditional masculinity norms may pose a challenge for men who are less likely to express their psychological vulnerabilities through internalizing symptoms or clear expression of weakness. This finding supports the idea that males may often mask their depression condition showing a wide range of alternative symptoms, in particular externalizing behavior [71,72]. In particular, a large body of research revealed that substance use, including smoking, during pregnancy is one of the most relevant associated factors with PPND [17,73,74] and should be considered as a fundamental aspect in the screening of early signs and symptoms of paternal affective disorder. Substance use disorder in new parents has been linked to adverse effects for parenting, which may compromise adequate caregiving. Research has widely documented the association between substance abuse and child negative outcome, including insecure attachment, maltreatment as well as emotional, behavioral, and health problems [75,76]. Moreover, in the group of “men at risk of externalizing behaviors”, hostility emerged as an important predictor to discriminate groups. Prior research highlighted the significance of the hostility, resentment, anger, and irritability as a relevant clinical manifestation of depression in men [33,77]. In this regard, it has been documented that irritability in men is associated with poor impulse control, anger attacks and aggression, substance misuse, and risk-taking or escape behaviors [78,79]. Hostility and substance use in fathers could also negatively affect parenting and couple relationships, leading to poor father–child interaction, aggressive parenting behaviors, and increasing the risk for engaging in intimate partner violence [80].

With respect to the second cluster defined as “psychologically distressed men”, we found that one father out of ten reported higher levels of depression and anxiety before childbirth. 

Interestingly, anxiety rather than depressive symptoms emerged as the most important predictor for this group. Evidence has shown that anxious symptoms during the perinatal period are common in men, suggesting the need to assess both depression and anxiety in expectant fathers [81]. A recent systematic review showed that the rates of anxiety disorders during the prenatal period ranged from 4.1% to 16% and remain substantially stable across the transition to parenthood [82]. This finding underlined that anxiety may be frequent in men who experience internalizing symptoms before childbirth, including those without significant depressive symptoms. Importantly, even in the case of men who experience internalizing distress, the assessment of depression could be limited, since anxiety is not adequately addressed. Both depression and anxiety in fathers have been associated with an increased risk for maternal and child health [81,83]. According to our results, fathers in this cluster could also show somatization symptoms experiencing the perception of physical dysfunction. This is consistent with previous studies showing that new fathers can express physical distress through somatic complaints and abnormal illness behaviors (the so-called Couvade Syndrome), which are considered to be part of the complex clinical picture of paternal perinatal distress [8,37]. 

Moreover, the association between the emerged psychological profiles and perceived stress was significant, with psychological health men reported a lower score in the scale of perceived stress than the other two clusters. Moreover, our results showed that psychologically distressed men reported higher perceived stress than the men at risk of externalized behavioral problems. According to previous studies, high perceived stress is associated to paternal affective disorders, especially with depressive and anxious symptomatology [3,26,27,84]. 

Finally, focusing on the association between the psychological profiles and marital adjustment, our findings revealed a significant relationship, with psychologically healthy men reporting the highest levels of marital adjustment and psychologically distressed men reporting the lowest levels. The lack of differences on dyadic adjustment between men at risk of externalized behavioral problems and psychologically distressed men, suggests that a poor intimate relationship is a common thread among men experiencing perinatal affective symptomatology. This result highlights the relationship between individual and couple’s functioning during pregnancy [11,25,85,86] and confirms the importance to consider dyadic and relational aspects as potential risk for men’s health both in case of externalizing and internalizing symptoms. Indeed, other authors have focused on the negative impact that perinatal affective disorders had on marital quality, especially on marital and sexual satisfaction [87,88,89].

Furthermore, in our sample, the presence of symptoms of psychological distress is not related to be a first-time father. Whereas some studies have revealed that multiparous parents exhibit a higher level of anxiety, depression symptoms, and a poor health-related quality of life than primiparous parents [18,19], others have reported that parity was unassociated with an increased risk of anxiety and depression or lower health-related quality of life scores during the perinatal period [27,90]. 

With the respect to the association between the psychological profiles and previous psychiatric disorders, our findings revealed a significant relationship, with psychologically distressed men reporting the highest percentage of previous psychiatric disorders compared with psychologically healthy men. These findings are consistent with previous studies that have identified the presence of previous psychiatric history related to the onset or the exacerbation of affective symptomatology during the perinatal period [84,91,92]. 

Similarly, the association between our psychological profiles and the presence of stressful life events in the preceding six months was statistically significant, with individuals of cluster “psychologically distressed men” having a higher percentage of stressful life events than the other two groups. This finding is supported by previous studies that identified the presence of stressful life events as a potential risk factor for perinatal affective disorders [20,27,85,93].

Our findings have relevant clinical implications. Prevention programs should be implemented including both parents from the prenatal period. Given that the quality of marital adjustment can be negatively affected by perinatal affective symptoms, a partner inclusive approach needs to be adopted throughout perinatal period [94]. For the screening and diagnosis, it is essential to consider the manifestation of externalizing behavior as depressive equivalents. We encourage extending the assessment by including non-traditional symptoms of perinatal affective disorder, following a gender-sensitive perspective. In this regard, it becomes crucial to raise the awareness of perinatal practitioners with respect to the clinical expression of paternal psychological distress. Fathers at risk of externalizing behavioral problems require a more in-depth diagnostic assessment, and a personalized treatment if needed. Interventions should be tailored to specific needs and clinical manifestations of the fathers, promoting partner reciprocal support.

## 5. Conclusions

The present study has strengths and limitations that should be addressed. This is the first pioneering study to examine the mental health of expectant fathers based on their levels of depression, anxiety, addiction, anger attacks/hostility and somatization by identifying psychological profiles. Second, in doing this, we also examined the association among these psychological profiles, perceived stress, and marital adjustment. Third, most of the studies on PPND have focused on first-time fathers and postnatal period, whereas we examined paternal mental health before childbirth, also including fathers with one or more children.

Despite these strengths, the findings of the present study should be interpreted with caution. Indeed, the cross-sectional nature of the data prevents us from drawing conclusions about causal direction. In the future, it could be useful for the research to implement a longitudinal design that makes it possible to expand the study to the postpartum period, analyzing the association between these psychological profiles and individual and couple variables during the postnatal period. Furthermore, it could be useful to anticipate the assessment during pregnancy to the first trimester. Indeed, data about prevalence rates of depression and anxiety and changes over time during the perinatal period vary widely [6,70]; thus, an early screening could make it possible to identify not just the presence of a symptomatology but also the trajectories of change over time [94]. Moreover, since our study was conducted on expectant fathers in their third trimester of partner’s pregnancy, it could be useful in the future to also obtain information on gestation weeks to assess if expectant fathers in their final weeks are at greater risk of PPAD than others. 

Another limitation of the study was to have few subjects with psychiatric history and stressful life events; future studies should better investigate the association between these variables and men at risk of PPAD.

Finally, we used self-report instruments that are not specifically developed to assess men’s perinatal distress. Future studies could include, for example, clinical interviews that can better capture the complexity and the variety of early signs of paternal perinatal affective symptomatology. Moreover, it is essential to develop new measures to evaluate a broad range of depressive equivalents increasing the sensitivity and specificity of the screening in the perinatal period. [1,8,37,41]. In this perspective, a team of researchers recently created the Perinatal Assessment of Paternal Affectivity (PAPA) [32,95] a self-report instrument for the screening of affective symptomatology in fathers based on recent research on perinatal affective disorders. This tool assesses different dimensions of paternal perinatal distress (anxiety, depression, irritability/anger, couple and relational difficulties, somatic complaints, risky behaviors, and addictions). Above all, an early diagnosis of Paternal Perinatal Affective Disorder (PPAD) may reflect a more comprehensive viewpoint to assess mental health of fathers during the perinatal period and avoid potential consequences on mothers’ mental health and children’s development [8].

In conclusion, our findings highlight the need to design an effective and also inclusive perinatal service for fathers’ psychological care, and they point out the importance of an appropriate gender-sensitive screening for detecting fathers’ affective symptoms given the impact of men psychological distress on the whole family well-being.

## Figures and Tables

**Table 1:** Sample’s descriptive characteristics.

(*n* = 350)	
Education	%
Elementary school	0.6%
Middle school diploma	12.2%
High school diploma	53.1%
Graduate degree	34.1%
Occupation	
Unemployed	0.9%
Student	1.5%
White/Blue collar	69.3%
Self-employed (professional/business owner)	26.9%
Executive/manager	1.2%
Marital status	
Married	50.6%
Cohabitant	49.4%
Number of children	
Primiparous	72.2%
Not Primiparous	27.8%
Stressful life events ^a^	
None	63.4%
One	32.4%
More than two	4.3%
Previous psychiatric diagnosis	
No	87.5%
Yes	12.5%

^a^ (job loss, serious financial problems, serious problems at work, divorce, mourning, family conflicts, fights, own illness, illness of loved ones).

**Table 2:** Descriptive statistics of our study variables.

	Mean	SD	Range
CES-D	8.13	4.95	0–30
ANX	2.10	2.65	0–17
SOM	3.48	4.02	0–29
HOS	1.63	2.44	0–17
PSS	10.97	5.66	0–30
DAS	124.47	15.27	0–151
Addiction/risky behaviors	%		
No	80.1%		
Yes	19.9%		

Note. CES-D, The Center for Epidemiological Studies Depression Scale; ANX, Anxiety; SOM, Somatization; HOS, Hostility; PSS, The Perceived Stress Scale; DAS, the Dyadic Adjustment Scale; SD, Standard Deviation.

**Table 3:** Bivariate correlations among the variables.

	CES-D	ANX	SOM	HOS	PSS	DAS
CES-D	1	0.575 **	0.447 **	0.450 **	0.551 **	−0.341 **
ANX		1	0.572 **	0.533 **	0.584 **	−0.274 **
SOM			1	0.386 **	0.365 **	−0.149 **
HOS				1	0.496 **	−0.354 **
PSS					1	−0.381 **
DAS						1

Note. CES-D, The Center for Epidemiological Studies Depression Scale; ANX, Anxiety; SOM, Somatization; HOS, Hostility; PSS, The Perceived Stress Scale; DAS, The Dyadic Adjustment Scale. ** *p* < 0.01.

**Table 4:** Cluster analysis: ANOVA and chi-squared test.

	Cluster		Error		F–χ^2^	Sig.
	Mean	df	Mean	df		
	Square		Square			
CES-D	1215.91	2	17.63	347	68.80	<0.001
ANX	683.85	2	3.16	347	215.71	<0.001
SOM	990.45	2	10.66	347	92.87	<0.001
HOS	371.1	2	3.90	347	95.14	<0.001
Addiction/risky behaviors		2			302.97	<0.001

Note. CES-D, The Center for Epidemiological Studies Depression Scale; ANX, Anxiety; SOM, Somatization; HOS, Hostility; df, degree of freedom.
